# MANDIBULAR OSTEITIS FIBROSA CYSTICA: AN UNUSUAL MANIFESTATION OF PRIMARY HYPERPARATHYROIDISM

**DOI:** 10.51894/001c.123081

**Published:** 2024-08-30

**Authors:** Gabriele Noreikaite

**Affiliations:** 1 Otolaryngology McLaren Oakland Hospital, Trinity Health Oakland Hospital

## 76

### INTRODUCTION

Osteitis fibrosa cystica (brown tumor) is an unusual manifestation of hyperparathyroidism secondary to imbalanced osteoclast activity. Disturbance in bone turnover can result in skeletal manifestations of hyperparathyroidism often seen as bony tumors of the long bones and vertebra. Involvement of the facial bones is uncommon with a prevalence of 0.1%. Due to improved screening techniques and frequent early detection of hyperparathyroidism, Brown tumors are quite rare. Nonetheless, brown tumors may develop in the absence of typical hyperparathyroidism symptoms causing delayed diagnosis. We present a case of undiagnosed primary hyperparathyroidism manifesting as a mandibular brown tumor.

### CASE DESCRIPTION

A 59-year-old female with a past medical history of diabetes mellitus presented to the emergency department for evaluation of right sided mandibular mass that had developed over the past two months. Maxillofacial CT scan showed a 4.2 cm right osseous lesion of the angle and body of the mandible. She was admitted for intra-operative biopsy with OMFS. Routine blood work was obtained, and a calcium of 12.9 (8.6-10.3 mg/DL) was noted. Further investigation with a PTH level showed 736 (12-88 pcg/mL). The patient was asymptomatic without complaints of polyuria, nausea, weakness, bone pain, hallucinations, etc. ENT was consulted and a sestamibi scan was ordered showing increased uptake within the left lower thyroid lobe. The patient underwent a parathyroid exploration for a suspected parathyroid adenoma. This was successfully removed with a post-operative PTH of 42.4. Biopsy of the mandibular lesion resulted the following day showing brown tumor of hyperparathyroidism.

### DISCUSSION

Asymptomatic primary hyperparathyroidism has a predilection for middle-aged women within the first decade of menopause. Common screening symptoms such as polyuria, fatigue, altered mental status, and bone pain may be absent placing this patient population at risk for developing osteoporosis and renal failure secondary to hyperparathyroidism. Uncommon presentations of hyperparathyroidism such as facial skeletal bony growths should not be ignored and warrant further investigation with a BMP, CBC, Ca level, PTH level, and biopsy. Appropriate early diagnosis of a brown tumor is vital for treating the underlying hyperparathyroidism. In turn, this helps prevent its complications.

